# The role of scarcity promotion and cause-related events in impulse purchase in the agricultural product live stream

**DOI:** 10.1038/s41598-023-30696-8

**Published:** 2023-03-07

**Authors:** Xiaolin Li, Mengqian Guo, Dunhu Huang

**Affiliations:** Business and Tourism School, Sichuan Agricultural University, Chengdu, 611830 China

**Keywords:** Human behaviour, Psychology, Environmental social sciences

## Abstract

Harvest agricultural products are perishable. If they cannot be sold, there will be serious grain loss and food waste. It is an important issue related to human sustainable development and urgent to address. As the most popular way of shopping, live shopping has achieved remarkable achievements, while the existing research is relatively silent on how to promote the sales of agricultural products in the context of live streams. Based on S–O–R theory and dual-system theory, three studies investigated the intrinsic mechanism of consumers’ impulse purchase intention (IPI) in live streams. The results show that scarcity promotion (SP) and cause-related events (CRE) are positively correlated with consumers’ IPI through arousal and moral elevation. Interestingly, when SP and CRE are presented at the same time, the impact of CRE on IPI is no longer significant. Overall, the proposed model could be used to predict consumers’ willingness and provide the choice of marketing strategy to promote the sale of agricultural products, which has significant theoretical and practical value.

## Introduction

Food waste is an important issue related to global food security and human sustainable development^1^. According to the data from the Food and Agriculture Organization of the United Nations (FAOUN), about 1.6 billion tons of food are lost or wasted every year, as well as more than 6 billion pounds of fresh agricultural products can’t be sold^2^. One of the most important reasons is that the way of harvesting agricultural products for the consumers is obstructed. It is urgent to alleviate the sales dilemma of agricultural products as well as food waste.

With the vigorous development of e-commerce, more and more consumers and businesses conduct transactions with agricultural products on e-commerce platforms, such as live streaming platforms^3^. The live stream shows the characteristics of real-time interaction, immersion^4^, and high decentralization^5^, which conforms to the current downward trend of consumption. It has strong endogenous growth potential. In 2020, the live streaming e-commerce market size reached 961 billion yuan in China, with a year-on-year increase of 121.5%^6^. Affected by COVID-19, many farmers sold agricultural products through the live streaming platform^7^. Stakeholders engagement for sustainability reporting shows that the live streaming platform is effective in increasing farmers’ income^8^. During the COVID-19 period, more than 600 County heads participated in the agricultural assistance live stream launched by Alibaba, driving the increase of transaction volume of more than 6 billion yuan^9^; The “No meet concert” Caring for public welfare activity held by TaoBao live stream sold 760 thousand kilograms of agricultural products for farmers in just four hours; CCTV launched the “Thank you for Hubei Spelling Order” live stream to help farmers sold more than 100 million yuan of agricultural products in just four hours^9^. The high efficiency of live stream is compatible with the high requirements of agricultural products’ short life cycle on sales channels, providing a feasible sales channel for agricultural products.

Online shopping eliminates spatial limitations therefore it is more impulsive than offline shopping^10^. Live streams particularly stimulate instant consumer purchases^11^. Considering the impact of life cycle of products, we propose that impulse purchases in live streaming scenarios may be able to effectively alleviate such seasonal sale dilemma of agricultural products. The existing research on agricultural products mainly focuses on exploring the causes of agricultural product sales difficulties^12^ and how to optimize the agricultural supply chain^13,14^. The relevant research about how to promote the sales of agricultural products by stimulating consumers’ impulse purchase in live stream is still weak.

Price characteristics are a key factor in stimulating consumers to buy impulsively^15^. During live streams, discounted products are often sold within a limited time or quantity. What’s more, the sale dilemma of agricultural products is a social event that has aroused widespread concern. With respect to the role of policy aspects towards these challenges, the government or enterprises often purchases these agricultural products in batches to help farmers tide over the difficulties. At the same time, the media reports these cause-related events (CRE) to spread positivity. When consumers watch the agricultural live stream, on the one hand, they may be stimulated by scarcity promotion(SP), on the other hand, they may be affected by the CRE. Both of them may arouse consumers’ impulse buying intentions. In marketing practice, SP and CRE marketing are commonly used by businesses^16,17^. Existing studies have explored the impact of SP on impulse purchases^16,18^, but few scholars have explored the impact of CRE and the interaction impact on impulse purchases. The main purpose of this study is to explore the impact mechanism of SP and CRE on consumers’ impulse purchase intention (IPI) and how SP and CRE interact to affect IPI. Moreover, provide operable suggestions for agricultural product sales. Our Study limits live stream products to agricultural products. Moreover, chose CRE as perceptual factors which is highly related to the characteristic of agricultural products. This increases the practicability and novelty of this Study.

## Theoretical background

### Agricultural product retailing

Agricultural products have the characteristics of a short life cycle and easy decay, their freshness decreases with time^19^, so it is required to complete the sales quickly in a short time. Poor sales channels and market saturation will hinder the sales of agricultural products^12^. E-commerce has become a new channel for the sales of agricultural products. The sales model of “Live stream + Agriculture + E-commerce” has played a good role in increasing the transaction and building brand reputation^20^. Consumers put forward higher and higher requirements for online shopping for agricultural products. The quality, online Word-of-Mouth, and logistics service will affect consumers’ willingness to buy agricultural products online^21^. Freshness is a critical feature to distinguish agricultural products. Due to the logistics cycle of online shopping, the freshness of agricultural products between buyer and seller is asymmetric, which aggravates the difficulty of fresh agricultural product supply chain coordination^13^. Faster transaction and logistics speed can alleviate the asymmetric freshness to a certain extent and enhance consumers’ intention to buy agricultural products online^14^. With the change in consumption concept, most consumers prefer to buy products with moral appeal^22^. The moral attribute of agricultural products will improve consumers’ acceptance and promote purchase^23^. Negative facial expressions in advertisements for poverty alleviation agricultural products can trigger higher purchase intention by evoking consumers’ greater guilt^24^.

### S–O–R Theory

The S–O–R theory originally came from the field of environmental psychology, which believes that the environment, as an external stimulus (S), further affects people’s external behavioral responses (R) by affecting people’s internal body state (O)^25^. Intrinsic body state (O) is divided into cognitive and affective responses, in which affect includes pleasure, arousal, and dominance. Extrinsic behavioral responses are divided into approach and avoidance responses^12^. The S–O–R model is widely used to explain online consumer behavior^26^.

In existing consumer behavior research, social presence^27–29^, website attributes^30–32^, promotion strategies^30,31^, visual attractiveness^28,33^, interpersonal relationships^33,34^ etc. were regarded as Stimuli (S). In the same way, attitudes^30,33,35^, emotions^32,36^, self-determination^37^, positive emotions^31,38,39^ were regarded as internal body states (O). It is worth mentioning that in addition to the above positive emotional states, some scholars also regarded fatigue and regret as the internal state (O) to probe how social media information overload affects users’ non-continuous use intention^26^.

In studies using the S–O–R model as a theoretical model framework, purchase^30,35,36^, impulse purchase^27,31,33,38,40^, repurchase^28^ willingness or behavior are often used as an organismal response variable (R). In addition, the willingness to use APP^29^, to participate in the online community^41^, and to like^34^ has also been widely studied.

Some scholars who conducted a research review found that the S–O–R framework is the most popular theoretical method to study online impulse buying^10^. The S–O–R model provides a visualized framework for studying the impact of marketing stimuli on consumers’ IPI and the internal psychological mechanism. Therefore, we rely on the S–O–R model as a central framework. In this study, we selected the SP and CRE as external stimuli(S). The arousal and moral elevation as internal body states (O) to explore the influence mechanism of impulse buying intention (R).

### Dual-system theory

Social psychologists believe that people’s thinking and decision-making involve two systems: perceptual and rational. The former makes a quick heuristic intuitive response (System 1), which is mostly affected by emotion, situation, and experience. On the contrary, the latter pays attention to the analytical thinking of logical reasoning (System 2)^42^. When making a decision, System 1 will quickly make a heuristic intuitive response, and then System 2 will conduct an analysis, which may modify the initial judgment^42^. But usually, the final choice is highly consistent with the initial response. Only in a few cases (such as after long-term reasoning), people will modify their initial choice^43^. Some scholars have proposed that the initial response is determined by the absolute strength between heuristic intuition and logical intuition, the subsequent change is determined by the relative strength of both^43^. Decision-makers who face greater time pressure and cognitive resource depletion will provoke the greater expression of System 1 response. It means that a high degree of information processing fluency is more likely to cause an intuitive response^44^. When the decision scene is integrated with the decision task, it will improve decision fluency and reduce consumers’ delayed-choice inclination^45^. When considering whether to buy an attractive single commodity, consumers may arouse an impulse. At this time, they will not activate System 2 to weigh the advantages and disadvantages^44^.

The cause-related events often affect people’s behavior by stimulating people’s moral emotions, and activate System 1 to make rapid emotional response. However, scarcity promotion involves limited-time, limited-quantity and discount calculation, which requires in-depth analysis and thinking with System 2. Previous studies have shown that abstract digital attributes such as prices are unlikely to cause an intuitive response, which usually requires System 2 to conduct in-depth evaluation and processing^44^. However, causes evoke more intuitive emotions, which are related to System 1. In view of this, we select cause-related events as perceptual factors (System 1) and scarcity promotion as rational factors (System 2) to explore the intrinsic mechanism of consumers’ IPI in the agricultural live stream.

### Live stream and impulse purchase

A Live stream is a pattern of information delivery that can record and broadcast events in real-time^46^. Compared with traditional e-commerce, live stream has the characteristics of synchronization and authenticity that bring obvious advantages in product display^47^. It caters to consumers’ information needs and pleasure needs, and increases consumers’ sense of presence and shopping experience^48^. In recent years, live streaming has become a popular subject in academic research. With respect to the usage intention, social distance^49^, social interaction, and identity^46^ have been studied. With respect to the continued usage intention, personal factors such as personal innovation^50^, perceived value^50^, emotional participation^51^, and self-identity^52^ have been studied. Li et al.^47^ discussed how social factors and technical factors affect user stickiness from the perspective of a social system and a technical system. The results show that social factors and technical factors have a positive impact on user stickiness by affecting anchor attachment and platform attachment respectively. Live stream requires a high degree of user participation^53^. With respect to the viewer engagement, researchers have shown that user-perceived value^54^, anchor response^11^, personalization of live streaming content^11^, and relationship identity^55^ all affect engagement.

Impulse purchase behavior is a sudden and hedonistic complex purchase behavior^56^, accompanied by strong and lasting impulse, which usually lacks information processing and a cognitive response process^57^. Previous studies have shown that consumers are more likely to buy impulsively in the online shopping environment^58^. Existing studies on impulse buying behavior mostly focus on online shopping environment (such as social media, community group buying, live streams, etc.), exploring the impact of flow^59–61^, quasi-social interactions^60–62^, price characteristics^15^, and product attractions^33,62,63^ on consumers’ impulse buying. Especially, in live streams, the atmosphere clues^64^ and the product recommendation of the anchor^58^ will stimulate consumers’ impulse purchases. However, no scholars have explored the interaction between perceptual and rational factors on consumers’ impulse purchase.

Through the above literature review, it is found that the existing research still lacks empirical research on combining agricultural products with the live streaming situation. Based on S–O–R theory and dual-system theory, we intend to promote the sales of agricultural products by studying IPI in live streams.

## Research model and hypothesis development

### Scarcity promotion, arousal, and impulse purchase intention

In live shopping, discounted goods are usually promoted through limited availability, either in terms of time or quantity. If the quantity or time exceeds the limit, the goods can’t be purchased at a discounted price. This promotion method is called scarcity promotion, which is specifically divided into limited-time promotion(LTP) and limited-quantity promotion(LQP)^18^. LTP refers to the sale of products or services at a discount price within a specified time. If the time is exceeded, the original price will be restored. Limited quantity promotion means that only a limited number of commodities or services are sold at a discount price. If the quantity exceeds the limit, you will not be able to enjoy the discount^16^. Information about SP in the live streaming room provides an urgent situation that urges consumers to make decisions in a short time. In urgent situations, consumers are more likely to rely on emotion to make decisions, which is accompanied by an increase in arousal level^18^. Arousal is the foundation of emotion and behavior^65^. The increase of arousal level is expressed as excitement or stimulation^66^. Previous studies have suggested that SP increases arousal by providing consumers with competitive situations. The higher the scarcity, the stronger the arousal^18^. Therefore, we propose the following assumptions:

#### H1a

Compared with no promotion (NP), LTP can lead to consumers’ stronger arousal.

#### H1b

Compared with no promotion (NP), LQP can lead to consumers’ stronger arousal.

In the live stream, the scarcity brings strong time pressure to consumers. This reduces their cognitive processing, which coincides with the concept of “impulse”^16^. SP gives consumers a feeling of “buying is earning” and “It’s now or never”, which inspires purchase impulse^16^. Considering that the original price will be restored when beyond the limited time or limited quantity, consumers may often ignore the feeling of regret post their impulse purchases^67^. Previous studies have shown that SP is a key stimulus in the pre-purchase evaluation stage of consumers’ impulse purchases^16^. The stronger the scarcity, the more it can stimulate consumers’ IPI. However, the effect of LTP and LQP is not always equal^68–70^. Aggarwal et al.^68^ considered that LQP was more effective than LTP on consumers’ purchase intention. Song et al.^70^ also proved that providing social presence information will strengthen consumers’ competitive awareness and promote the impact of LQP on consumers’ purchase intention. Agricultural product live stream is a live activity to help the sales of agricultural products. The activity itself shows that there are difficulties in the sale of agricultural products, that is, the overall quantity of agricultural products is plentiful. This weakens the impact of quantity restriction on IPI in live stream, and reduces the difference between LQP and LTP. Therefore, we propose the following assumptions:

#### H2a

Compared with NP, LTP can provoke consumers into stronger IPI.

#### H2b

Compared with NP, LQP can provoke consumers into stronger IPI.

#### H2c

There is no significant difference between LTP and LQP on consumers’ IPI.

### The mediating effect of arousal

In the online shopping environment, arousal is an excited emotional state stimulated by the online cues^18^. The competition arousal model assumes that the competition or time pressure felt by consumers will stimulate arousal^71^. The increase in arousal level will limit attention to the most critical clues, so make it is easy to ignore more product information, accompanied by less careful consideration in decision-making^71,72^. Previous studies have shown that consumers’ arousal positively affects impulse buying^18^. SP brings time pressure to consumers, increases arousal, and reduces cognitive thinking^59^. We consider that SP evoke IPI. It is due to the rise of arousal. Based on the above logic, we assume that:

#### H3

Arousal plays an intermediary role in the impact of SP on IPI.

### The mediating effect of moral elevation

Cause-related event is a concept related to Cause-related Marketing. We explain a cause-related event as an event related to the ethical actions made by the government, enterprises, or the public to help sell agricultural products. This may not be beneficial to them but can improve the overall social welfare level. When enterprises devise advertisements, adding cause-related information will lead to a more favorable consumer response^73,74^. The moral behavior of others will stimulate individuals’ moral elevation^75,76^. Moral elevation refers to the feeling of warmth, movement, or chest fever generated by individuals when they see the moral behavior of others^75^. It further stimulates individuals to show prosocial behaviors such as donation or participation in voluntary activities^77^. People who watch videos about others’ moral behaviors will be more eager to have closer contact with good-doers, which makes them willing to actively participate in prosocial behaviors^78^. We hold that the impulsive purchase behavior of agricultural products is consistent with prosocial behavior. Previous studies have shown that moral elevation plays an intermediary role between corporate social responsibility activities and prosocial behavior^77,79,80^. Therefore, we assume that:

#### H4a

CRE have a significant positive impact on consumers’ IPI.

#### H4b

Moral elevation plays a mediating role between CRE and IPI.

### Interaction between cause-related events and scarcity promotion

Discount promotions and cause-related marketing are the common marketing measures. Some studies have shown that consumers have more positive attitudes toward the advertisements embedded with cause-related marketing information^73^. When cause-related marketing is combined with price discounts, there is an inverted U-shaped relationship between them. Appropriate discounts work best. Deep discounts paired with cause-related marketing will weaken the warm feeling consumers get from participating in cause-related marketing activities^74^. In the labor market, when social preference is the main motivation, the external monetary stimuli dilute the signaling value of prosocial behaviors, potentially creating a “crowding-out effect”^81^. In view of this, we consider that the simultaneous occurrence of price discounts and causative events may not simply equal the sum of their individual effects on consumers.

Self-signal theory suggests that when people engage in prosocial behaviors, they not only convey positive signals to others but also to themselves, reinforcing the individual’s helpfulness image perception^82^. In the live stream, when consumers perceive CRE and SP at the same time, the external motivation caused by SP makes consumers doubt their helpful image. They need to expend more cognitive resources to balance, thus buffering immediate impulses. This paper argues that when SP and CRE appear at the same time, consumers are mainly driven by economic factors related to SP. CRE will not affect consumers’ IPI.

According to this, we assume that:

#### H5

SP and CRE interact to influence consumers’ IPI. Specifically, SP negatively moderates the impact of CRE on IPI.

**Figure 1:**
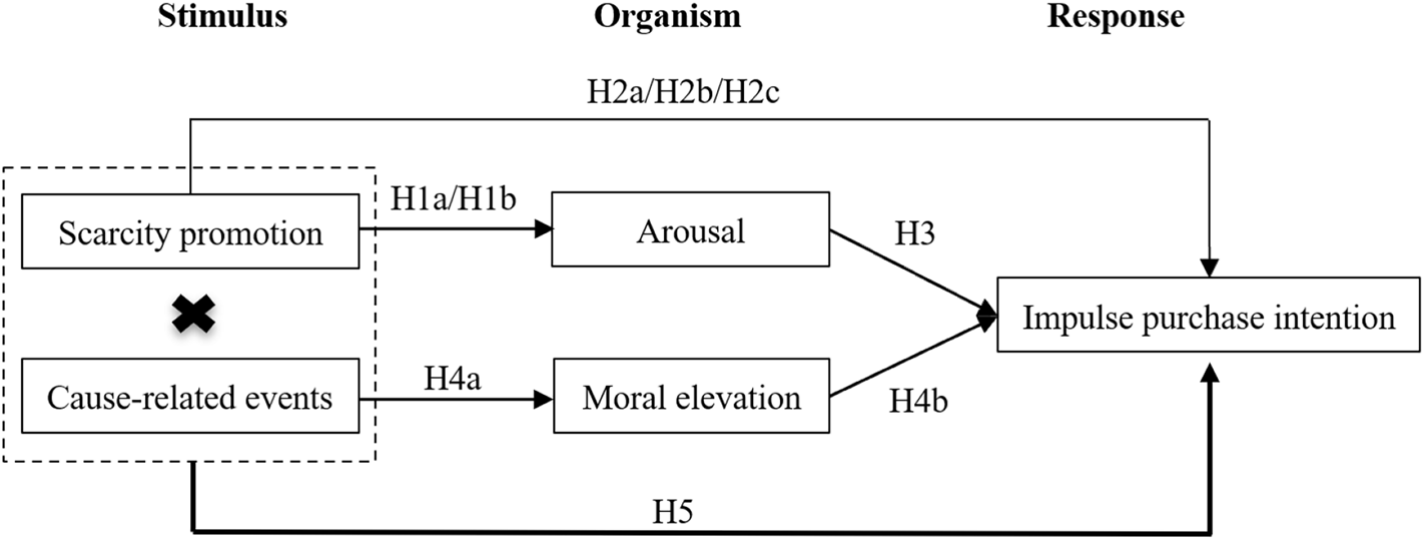
Theoretical model.

Based on the above research assumptions, the theoretical model is shown in Fig. 1.

## Overview of studies

This paper intends to test hypotheses through two experiments.

Experiment 1 tested how scarcity promotion and CRE lead to consumers’ IPI, that is, hypotheses H1–H4;

Based on repeatedly verifying the conclusion of experiment 1, experiment 2 focused on the interaction of CRE and SP on consumers’ IPI, that is, hypothesis H5.

## Study 1a

### Pretest

#### Purpose

Ascertain the limited time and quantity that have the same stimulating effect on participants.

#### Design

Based on the experimental design of Aggarwal et al.^68^, 10 graduate students who have watched the e-commerce live stream at a university in China were selected to participate in the pretest (male to female = 1:1). In the pretest, firstly, we asked participants the limited time that can bring the sense of scarcity when watching the live stream. Then we asked participants to answer the limited quantities that can bring the same sense of scarcity. In the end, more than half (8/10) of participants answered that the 5 min limited-time discounts make them feel scarcity. Therefore, we regarded 5 min as a limited promotion time. In addition, in the context of LQP, consumers will feel the competition. The scarcity perception is affected by the number of online viewers. In view of this, we also controlled the number of viewers. According to the relevant data of Taobao rural stream, the viewers of different rural streamers range from 20 to 100 thousand while most streams’ online viewers are about 50 thousand. Therefore, the material sets the number of online viewers to 50 thousand.

The specific situation of the pretest is “Suppose you are watching an agricultural live stream, with about 50 thousand people online. The products involve seasonal agricultural products such as grapes, litchis, onions et.al. All products are sold in groups, with the price of each group ranging from 30 to 50 yuan. Within the first five minutes of each product being sold, you can enjoy a 30% off for placing an order (automatic deduction for placing an order)”. After reading the experimental situation, all participants were asked to answer the number of limited promotion that brings the same scarcity feeling as a 5 min limited promotion. Then we calculated the mean and mode of all results. Some studies have shown that a 30% off is the critical level of consumer price perception^83^. Therefore, we set the price discount as 30%.

#### Results

All participants thought that the 5-min could feel scarce. The average of the corresponding was 1088.88 and the mode was 1000 (6/10). In live streams, the limited quantity is usually an integer. Therefore, we use 1000 groups as the limited quantity of discounted agricultural products for subsequent formal experiments.

### Method

#### Participants

In Study 1a, 118 subjects were recruited from Credamo, which is a professional online platform in China. After completing the experiment, each subject will receive 1 yuan as a reward. Subjects who haven’t watched the E-commerce live stream and failed to pass the screening were excluded. As a result, 98 valid samples (55.1% female, 32 in the LTP group, 32 in the LQP group, 34 in the NP group) were obtained. The demographic information of Study 1a is shown in Table 1. Informed consent was obtained from all participants.

**Table 1:** The demographic information of Study 1a.

Items		Frequency	Percentage(%)
Gender	Male	44	44.90
	Female	54	55.10
Age	18–25	50	51.02
	26–35	40	40.82
	36–45	6	6.12
	Over 45	2	2.04
Monthly disposable income	Under 1000	10	10.20
	1000–2000	21	21.43
	2001–3000	11	11.22
	Over 3000	56	57.15

#### Design and procedure

Study 1a adopts a single factor (LTP vs. LQP vs NSP) inter-subject experimental design. The purpose is to verify H1-H3, that is, the positive impact of SP on consumers’ IPI and the mediating role of arousal. Through the collection of information on agricultural products with blocked sales, the experimental materials selected the seasonal fruit—Kyoho Grapes. After investigating the market price, it was determined that the grapes were sold in groups, with 5 kg and 39 yuan per group. An impulse purchase is a sudden and unplanned purchase behavior, so the experimental situation is set as “You open Taobao to choose a T-shirt, and occasionally click into the benevolent live stream of Kyoho grapes with blocked sales…”. In order to dispel consumers’ concerns about the quality of grapes, the experimental materials also explained the blocked reasons with “Mature Kyoho grapes have sufficient glucose and compact fruit, but it faces the salable dilemma due to its single sales channel.” The LTP in the experimental materials was set as “The discounted time of grapes in the live streaming room starts from being put on the shelf. In the first 5 min, you can enjoy 30% off for placing an order (automatic deduction for placing an order). But orders exceeding the limited-time need to be purchased at the original prices”. The LQP was set as “The discounted grapes in the live streaming room are counted from being put on the shelf. The first 1000 pieces can enjoy 30% off (automatic deduction for placing an order). While from the 1001st piece it needs to be purchased at the original prices”. The control group is without promotion information. In order to avoid the impact of real events on the experimental results, the experimental materials used “an ecological vineyard in a village” instead of grape origin. The number of live online viewers in all scenarios was 50 thousand. All methods were carried out in accordance with relevant guidelines and regulations. The study was approved by the Sichuan agriculture University Human Research Ethics Committee.

#### Measure

The scale used in the experiment is appropriately modified based on the maturity scale to accord with the context of this study. The IPI scale refers to the scale prepared by Beatty and Ferrell^84^, including three items (Cronbach’s = 0.939) such as “There is a great possibility to buy Kyoho grapes in the live stream, although I didn’t want to buy them before”. The arousal scale refers to the scale compiled by Russell and Mehrabian^85^, which includes three items: “The discount information provided by the material makes me feel ‘excited’, ‘stimulated’ or ‘Aroused’” (Cronbach’sα = 0.918). The perception of SP is measured by “In this live stream, I feel that the time (quantity) that I can enjoy discount is limited”, “In this live stream, I feel that if I buy within the promotion time (quantity), I can save more money”. Considering the attractiveness of grapes described in the experiment to different subjects, personal price awareness, and monthly disposable income may disturb the result, we also measured them. Product attractiveness is measured by “Kyoho grapes in the material are attractive to me”. Personal price awareness refers to the scale^86^ of LICHTENST et al., which includes three items such as “I don’t want to spend extra energy looking for a lower price” (Cronbach’sα = 0.887). On the other hand, we hold that personal self-construal will affect the results. Self-construal represents how individuals define themselves and perceive their personality^87^. It is divided into independent self-construal and interdependent self-construal. People with an independent self-construal pay more attention to personal goals rather than effective social relations. On the contrary, people with an interdependent self-construal pay more attention to the goals of others^88^. Self-construal was measured with reference to Choi and Totten’s scale^89^ (Cronbach’s α independent = 0.742, Cronbach’s α interdependent = 0.800). The following self-construal coefficients were constructed: (interdependent-independent)/(interdependent + independent)^90^. All scales adopt Likert 7-point scale (1 = “strongly disagree”, 7 = “strongly agree”). The complete scale is provided in the appendix (Supplementary Information 1).

### Results and discussion

#### Manipulation check

The manipulation check of SP was successful (Compare with absolute standard 4) (M_LTP_ = 6.09, t (31) = 16.63, *p* < 0.001; M_LQP_ = 6.27, t (31) = 21.05, *p* < 0.001). Harman’s one-factor test showed that the percentage of the first principal component in the cumulative total variance is less than 40%, so there was no serious common method variance.

#### Hypothesis testing

Firstly, we estimated the proposed main effects of SP. Independent samples t-test showed that there were significant differences in SP groups. In LTP group, M _LTP_ = 5.52, M _NSP_ = 3.67, t (64) = − 4.89, *P* < 0.001; In LQP group, M _LQP_ = 5.81, M_NSP_ = 3.67, t (64) = − 6.66, *P* < 0.001. This showed that SP could promote consumers’ IPI, supporting H2a, H2b. Further, the independent samples t-test was conducted for the LTP group and the LQP group. The results indicate that there is no significant difference (M_LTP_ = 5.52, M _LQP_ = 5.81, t (62) = − 0.93, *P* > 0.05).

Next, the mediating effect of arousal was estimated by Bootstrap^91^. Firstly, we tested the mediating effect of the LTP. The results showed that LTP played a positive role in consumers’ arousal (β = 0.731, *P* < 0.001), supporting H1a. The LTP had a positive impact on IPI. The total effect was 0.638 (t (65) = 2.57, *P* < 0.05), supporting H2a. The mediating effect of arousal was significant (95%, LLCI = 0.070, ULCI = 0.742). The indirect effect was 0.380. After controlling the mediate variable, the direct impact of LTP was not significant (95% CI = [− 0.157, 0.673], *P* > 0.05). That is, arousal played a complete mediate role between LTP and IPI, supporting H3. Similarly, the mediate effect test was carried out for the LQP. The results showed that LQP exerted a positive impact on consumers’ arousal (β = 0.281, *P* < 0.001). The H1b was supported. It exerted a positive impact on IPI too, with a total effect of 0.325, t (65) = 2.48, *P* < 0.05, supporting H2b. The mediating effect of arousal was significant (95%, LLCI = 0.009, ULCI = 0.266, indirect effect b = 0.111). After controlling arousal, the influence of the LQP on IPI wasn’t significant (95% CI − 0.032, 0.460, *P* > 0.05). It meant that arousal played a complete mediate role between LQP and IPI. The H3 was supported. The specific regression coefficients are shown in Tables 2, 3.

**Table 2:** the mediating effect of arousal (LTP).

Variable	Impulse purchase intention		
	Total effect	Mediating effect	Direct effect
Covariate			
Price awareness	0.080	− 0.038	0.119*
Self-construal coefficients	0.822	− 0.004	0.826
Product attractiveness	0.861***	0.361***	0.499***
Monthly disposable income	0.041	0.032	0.008
Independent variable			
Limited-time promotion	0.638*	0.380*	0.258
Mediator			
Arousal			0.519***
R^2^	0.794	0.662	0.871

*, **, ***In the table represent *P* < 0.05, 0.01 and 0.001 respectively.

**Table 3:** The mediating effect of arousal (LQP).

Variable	Impulse purchase intention		
	Total effect	Mediating effect	Direct effect
Covariate			
Price awareness	0.068	0.013	0.055
Self-construal coefficients	0.715	0.111	0.603
Product attractiveness	0.756***	0.223***	0.533***
Monthly disposable income	0.115	0.077	0.038
Independent variable			
Limited-quantity promotion	0.325*	0.111*	0.214
Mediator			
Arousal			0.393***
R^2^	0.793	0.667	0.831

*, **, ***In the table represent *P* < 0.05, 0.01 and 0.001 respectively.

### Conclusion

Study 1a tests how SP impact IPI. The results show that SP (LTP and LQP) can promote consumers’ IPI by stimulating arousal. Moreover, arousal plays a complete mediate role. The hypothesis H1–H3 is supported. Study 1a also examines there is no significant difference between LTP and LQP on IPI. Next, study 1b will test the impact of CRE on impulse buying and its internal mechanism.

## Study 1b

### Method

#### Pretest

The purpose of the pretest is to determine the CRE used in the experiment. We investigated the blocked reasons for agricultural products and collected cause-related news. At last, we extracted “a special live stream of Kyoho grapes with blocked sales”, “charitable project for farmers”, “ to solve the sale problem, a local enterprise sent its vehicles to purchase the first batch of mature grapes as employee benefits” as information of CRE.

#### Participants

In Study 1b, 75 subjects were recruited through Credamo to participate in the experiment. After completing the experiment, each subject will receive a reward of 1 yuan. Finally, we got 68 valid samples (63.2% female, CRE 31 and without CRE 37). The demographic information is shown in Table 4. Informed consent was obtained from all participants.

**Table 4:** The demographic information of Study 1b.

Items		Frequency	Percentage(%)
Gender	Male	25	36.80
	Female	43	63.20
Age	18–25	62	91.20
	26–35	6	8.80
	36–45	0	0
	Over 45	0	0
Monthly disposable income	Under 1000	11	16.18
	1000–2000	28	41.18
	2001–3000	7	10.29
	Over 3000	22	32.35

#### Design and procedure

Study 1b adopted a single factor (cause-related event: yes vs. no) inter-subject experimental design. The purpose is to test hypothesis H4. The materials provided information on CRE. All methods were carried out in accordance with relevant guidelines and regulations. The study was approved by the Sichuan agriculture University Human Research Ethics Committee.

#### Measure

The impulse purchase intention, arousal, personal price awareness, and self-construal were the same as in Study 1a. The moral elevation refers to the scale^79^ prepared by Jiang and Zheng. The scale is divided into three dimensions, including 11 items such as “all sectors of society make an effort to help farmers, it makes me moved” (Cronbach’s α = 0.969). The scale has high internal consistency. The complete scale is provided in the appendix.

### Results and discussion

#### Manipulation check

The manipulation test of CRE showed that the manipulation was successful (Compare with absolute standard 4) (M _cause event group_ = 5.48, t (30) = 7.366, *P* < 0.001).

#### Hypothesis testing

First, we estimated the main effect of CRE on IPI. The independent sample t-test results showed that CRE can stimulate consumers’ IPI (M_experimental group_ = 4.688, M _control group_ = 3.721, t (66) = − 2.801, *P* < 0.01). The H4a was supported.

Next, the Bootstrap was used to examine the mediate effect of moral elevation^91^. The results showed that CRE had a positive impact on consumers’ moral elevation (β = 0.788, *P* < 0.001). The mediating effect of moral elevation was significant (indirect effect b = 0.335, 95% CI = [0.106, 0.603]). After controlling the mediate variables, the direct impact of CRE on IPI wasn’t significant (95% CI = [− 0.309, 0.521], *P* > 0.05). It meant that moral elevation played a complete mediate role between CRE and IPI. The H4b was supported. The specific regression coefficients are shown in Table 5.

**Table 5:** The mediating effect of moral elevation.

Variable	Impulse purchase intention		
	Total effect	Mediating effect	Direct effect
Covariate			
Price awareness	0.125	0.055	0.070
Self-construal coefficients	0.359	− 0.333	0.692
Product attractiveness	0.742***	0.180***	0.562***
Monthly disposable income	0.019	− 0.066	0.085
Independent variable			
Cause-related events	0.441*	0.335***	0.106
Mediator			
Moral elevation			0.425***
R^2^	0.704	0.505	0.765

*, **, *** in the table represent *P* < 0.05, 0.01 and 0.001 respectively.

### Conclusion

Study 1b examines the impact of CRE on impulse buying. The results show that CRE promote consumers’ impulse buying by moral elevation. The moral elevation plays a complete mediate role. Hypothesis H4 was supported.

Study 1 examines the effects of SP and CRE on consumers’ IPI. The results show that both CRE and SP can stimulate IPI. In marketing practice, businesses often integrate a variety of marketing methods to achieve better marketing effects. Studies have shown that strategies that combine intrinsic motivation with extrinsic motivation are not always effective^81^. Next, we will explore the interactive effects of SP and CRE on consumers’ IPI.

## Study 2

### Method

#### Participants

After screening the samples, 124 valid samples were obtained (Female = 62.9%, 31 CRE * SP, 31 CRE * NP, 30 no CRE * SP, 32 no CRE * NP). After completing the experiment, each subject will receive a reward of 1 yuan. The demographic information of Study 2 is shown in Table 6. Informed consent was obtained from all participants.

**Table 6:** The demographic information of Study 2.

Items		Frequency	Percentage (%)
Gender	Male	46	37.1
	Female	78	62.9
Age	18–25	45	36.3
	26–35	66	53.2
	36–45	8	6.5
	Over 45	5	4.0
Monthly disposable income	Under 1000	5	4.0
	1000–2000	26	21.0
	2001–3000	24	19.4
	Over 3000	69	55.6

#### Design and Procedure

Study 2 adopted a 2 (CRE: yes vs. no) * 2 (SP: yes vs. no) between-subject experimental design. The purpose is to measure the interactive effect of CRE and SP on consumers’ IPI. In order to improve the external validity of the experiment, Study 2 used purple garlic as the agricultural product material. In Study 2, “Alibaba platform subsidizes all discounts” was added to highlight the CRE. LTP and LQP were simultaneously presented, nevertheless, with different discounts in different periods. All subjects were randomly divided into four groups. The experimental procedure and measurement scale were the same as in Study 1. All methods were carried out in accordance with relevant guidelines and regulations. The study was approved by the Sichuan agriculture University Human Research Ethics Committee.

### Results and discussion

#### Manipulation check

The manipulation of CRE in Study 2 was successful (M_CRE_ = 5.68, t (61) = 14.10, *P* < 0.001). The manipulation of SP in Study 2 was successful (M_SP_ = 0.5.90, t (60) = 20.05, *P* < 0.001). The data passed the Harman common method bias test^92^. There was no serious common method bias problem.

#### Hypothesis testing

Firstly, we analyzed the main effect of CRE on subjects’ IPI. The scores of CRE were significantly higher than without CRE (M_CRE_ = 5.350, M no _CRE_ = 4.307, t (122) = − 3.885, *P* < 0.001). Then the main effect of SP was tested. The results showed that SP played a positive role on consumers’ IPI (M_SP_ = 5.383, M_NSP_ = 4.291, t (122) = − 4.089, *P* < 0.001).

**Figure 2:**
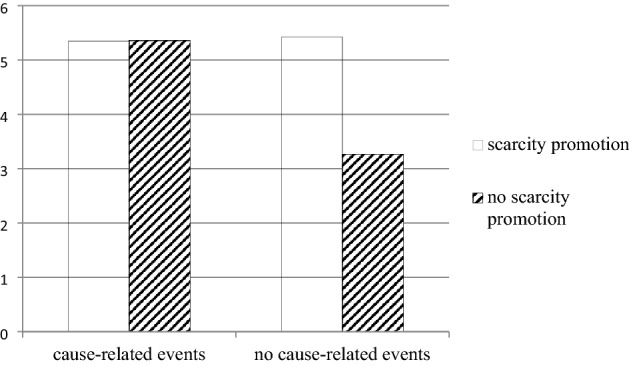
Interaction effect of SP and CRE on IPI.

Then, we adopted the covariance analysis. The price attractiveness, price awareness, and self-construal were regarded as covariates. The results showed that the main effect of CRE was significant (F (1117) = 28.303, *P* < 0.0005). The main effect of SP was also significant (F (1117) = 10.906, *P* < 0.05). The H1 and H2 have been verified again. The interaction between CRE and SP on IPI was significant (F (1117) = 13.870, *P* < 0.0005). In SP group, M_CRE_ = 5.344, M no_CRE_ = 5.422, t (59) = − 0.296, *P* > 0.05. In NSP group, M_CRE_ = 5.355, M no_CRE_ = 3.260, t (61) = 5.531, *P* < 0.001. That is SP moderates the impact of CRE on an IPI. Without scarcity promotion, CRE have a greater positive impact on IPI. The H5 was supported. The interaction effect is shown in Fig. 2.

### Conclusion

An impulse purchase is an immediate purchase behavior aroused by external stimuli^16^, which usually undergoes less cognitive processing. According to the motivational crowding theory, if the SP and CRE are presented to consumers at the same time, the external motivation caused by promotion will cover up or even destroy the internal motivation caused by the CRE^93^. It requires consumers to expend more cognitive resources to balance the conflict between an individual’s positive image cognition and bargain hunter image. The results of Study 2 show that SP and CRE would interact to affect consumers’ IPI. When adding SP, consumers are mainly driven by promotional incentives. At this time, CRE will no longer significantly affect consumers’ IPI. When without SP, the CRE play a significant positive impact on consumers’ IPI.

## General discussion

Based on S–O–R theory and dual-system theory, combined with the competitive arousal model and self-signal theory, this study discussed how scarcity promotion and cause-related events lead to impulse purchase intention in the live stream.

This paper verifies the proposed hypotheses through two experiments in the background of agricultural product live stream. Study 1 consists of 1a and 1b, which respectively explore the impact of scarcity promotion and cause-related events on impulse buying. Study 1a verifies that scarcity promotion promotes consumers’ impulse purchase intention by stimulating consumers’ arousal, in which arousal plays a complete mediate role. Scarcity promotion provides consumers with an urgent situation, the perceived time and competitive pressure will increase consumers’ arousal, limit the distribution of attention, and make it easier to buy impulsively. Study 1b verifies that the cause-related events can enhance consumers’ impulse purchase intention by provoking consumers’ moral elevation, in which the moral elevation plays a complete mediate role. The moral elevation is a warm feeling after witnessing moral beauty (such as charity, loyalty, self-sacrifice, etc.)^75^. It will lead to subsequent prosocial actions. After reading the materials that other groups in society have made efforts to solve the sales problem, intense moral elevation leads to impulse purchase intention.

Based on the conclusions of Study 1, Study 2 further explores the interaction between scarcity promotion and cause-related events. There are interaction effects. When scarcity promotion is added, the impact of cause-related events on consumers’ impulse purchase intention is in a secondary position^81^. Cause-related events no longer significantly affect consumers’ impulse purchase intention. Adding scarcity promotion may make consumers feel that they don’t want to do a good deed, but enjoy discounts^74^. It results in self-doubt, which weakens consumers’ impulse to buy agricultural products. When providing just cause-related events, consumers are only stimulated by a single stimulus, which is more likely to produce a direct-purchase impulse. So it has a significant positive impact on consumers’ impulse purchase intention.

## Theoretical and practical implications

### Theoretical implications

Firstly, this paper extends the research vision to cause-related events and enriches elements affecting consumers’ impulse purchases in the live streaming context. In the past, scholars have studied impulse purchase under the live streaming scenario, focusing on factors such as platform technical characteristics^47^, atmosphere clues^64^, and social existence^94^. However, few scholars have considered cause-related events as stimulating factors. There are some differences between cause-related events and cause-related marketing. The cause-related events emphasize the good deeds made by the third-party groups except for the purchasing merchants. Nevertheless, cause-related marketing refers to the merchants’ promise to donate money to support the public welfare for each unit of goods sold, so as to improve product transactions^95^. This study provides a new perspective on cause-related marketing.

Secondly, this paper introduces the moral elevation in the field of psychology into Marketing Management, which enriches the connotation of S–O–R theory. The S–O–R theory is widely used in consumer behavior, the internal body state (O) involves consumers’ cognition and emotion^18^^,^^30^^,^^31^. But no scholars have considered moral elevation as an emotional response to explore its impact on the external behavior response (R). Moral elevation involves four parts: emotion, body, cognition, and behavior^96^. It is consistent with the connotation of internal body state in S–O–R theory. We expand the scope of application of the S–O–R theory.

Finally, we consider the impulse purchase of blocked agricultural products as prosocial behavior, which promote the integration of Marketing Management and Social Psychology. Previous studies have shown that perceiving the importance of cause-related events will significantly improve consumers’ attitudes towards enterprises^97^ and purchase intention^98^. The moral elevation will stimulate individuals to make prosocial behaviors, such as donation, volunteering, etc. However, few scholars have meditated that prosocial behavior may be an impulse. This paper first makes an attempt to consider impulse behavior as a prosocial behavior. It deepens the scope of prosocial behavior in the field of Marketing Management.

### Practical Implications

The conclusion provides some enlightenment for agricultural products retailing in the live streaming environment. Some suggestions are put forward for stakeholders’ engagement for sustainability.

First, businesses can adopt limited-time or limited-quantity promotion strategies to enhance consumers’ emotional arousal and impulse purchase intention. Information asymmetry is a key factor to obstruct agricultural product sales. Live streaming realizes the rapid circulation of information. It alleviates the sales dilemma caused by information asymmetry to a certain extent. When consumers watch the live stream, they are more vulnerable stimulated by scarcity promotion, resulting in emotional arousal. In marketing practice, businesses should choose live streams as the sales channel for agricultural products. What’s more, It is necessary to adopt scarcity promotion in a live stream to create a sense of urgency.

Second, businesses should expose that social groups are endeavoring to alleviate agricultural products’ dilemma. For example, provide some relevant news by expressing gratitude or updating the current blocked situation of agricultural products in the live streaming preview. The anchor should read the encouraging reviews and update the real-time sales, so as to stimulate the moral elevation of other consumers in the live streaming room and stimulate impulse purchase intention.

Third, businesses should choose to show either cause-related events or scarcity promotion. Adding scarcity promotion and cause-related events can’t get the superimposed marketing effect. On the contrary, it is counterproductive and dilutes consumers’ purchase impulses. In marketing practice, businesses should choose appropriate information to cause consumers’ purchase impulse and achieve the best sales performance at the least cost.

Fourth, with respect to the role of policy aspects towards agricultural product sales dilemma, the government should guide enterprises to bear corresponding social responsibilities in the sale of agricultural products and provide policy preference and financial support. The media should report more cause-related events about agricultural products and carry forward the socialist core values. The e-commerce platform can set up a special area such as a special charitable live stream for poverty alleviation and agriculture assistance and update the information on agricultural products. The Blocked sales of agricultural products seriously hinder World’s agricultural and rural development. Carrying out supply-side reform and improving the quality of agricultural products is the only way for rural development. However, at the same time, to maintain a long life cycle, how to sell the harvested agricultural products at the regular market price and ensure farmers’ income is also a key issue.

## Limitations and further research directions

This study still has some limitations.

Firstly, considering the seasonality of harvested agricultural products, the immediacy of an impulse purchase, and the high efficiency of the live stream, we proposed to use impulse purchase in the live streaming situation to promote the sale of agricultural products. However, impulse purchase is temporary. Future research can consider how to promote repurchase.

Secondly, this paper measure consumers’ impulse purchase intention instead of actual impulse purchase behavior. In future research, field laboratory experiments can be set up to record the subjects’ actual impulse buying behavior.

Finally, this paper didn’t explore the boundary conditions. Future research can continue to explore other factors’ roles, such as the anchor type or anchor-product match-up.

## Supplementary Information

Supplementary Information.

## Data Availability

The datasets generated during or analyzed during the current study are available from the corresponding author on reasonable request.
