# Supplementary material for: The role of scarcity promotion and cause-related events in impulse purchase in the agricultural product live stream
Source: Sci Rep. 2023 Mar 7;13:3800. doi: 10.1038/s41598-023-30696-8 (PMC9990005; doi:10.1038/s41598-023-30696-8)
Supplement: Supplementary file 1 — Supplementary Information. [file 41598_2023_30696_MOESM1_ESM.docx]

**Appendix Summary of measurement items**

| **Variables** | **Sources** | **Measurement Items**  **(1 = Strongly disagree, 7 = Strongly agree)** | **Cronbach's α** |
| --- | --- | --- | --- |
| Impulse Purchase Intention | Beatty & Ferrell ,1998 | There is a great possibility to buy Kyoho grapes in the live stream, although I didn't want to buy them before | 0.939 |
|  |  | I have a strong desire to have this Kyoho grape, although I didn't want to buy it before |  |
|  |  | I want to buy this Kyoho grape right now, although I didn't want to buy it before |  |
| Arousal | Russell & Mehrabian, 1977 | The discount information provided by the material makes me feel excited | 0.918 |
|  |  | The discount information provided by the material makes me feel stimulated |  |
|  |  | The discount information provided by the material makes me feel Aroused |  |
| Personal price awareness | LICHTENST et al.,1993 | I don't want to spend extra energy looking for a lower price | 0.887 |
|  |  | I think the money saved by search at low price is usually not worth spending time and energy |  |
|  |  | I never browse multiple stores for low prices |  |
| Independent Self-construal | Choi & Totten's,2012 | It's important for me to keep active imagination | 0.742 |
|  |  | I would like to be different in many aspects |  |
|  |  | It's important for me to possess independent personality |  |
| Interdependent Self-construal | Choi & Totten's,2012 | The happiness of people around me is my happiness | 0.800 |
|  |  | I will sacrifice my own interests for the collective interest |  |
|  |  | I often feel that maintaining good interpersonal relationships is more important than obtain achievements |  |
| Moral Elevation | Jiang & Zheng,2017 | The society make an effort to help farmers, it makes me moved | 0.969 |
|  |  | The society make an effort to help farmers, it makes me appreciative |  |
|  |  | The society make an effort to help farmers, it makes me feel inspired |  |
|  |  | The society make an effort to help farmers, it makes me believe that the world is still beautiful |  |
|  |  | The society make an effort to help farmers, it makes me believe that the world is full of kindness and generosity |  |
|  |  | The society make an effort to help farmers, it makes me believe that man’s nature at birth is good |  |
|  |  | The society make an effort to help farmers, it makes me believe that most people's behavior is admirable |  |
|  |  | The society make an effort to help farmers, it makes me want to be a better person |  |
|  |  | The society make an effort to help farmers, it makes me comprehend how to become a better person |  |
|  |  | The society make an effort to help farmers, it makes me want to help others more |  |
|  |  | The society make an effort to help farmers, it makes me want to participate in this cause |  |
